# Neural Correlates of Morphological Processing: Evidence from Chinese

**DOI:** 10.3389/fnhum.2015.00714

**Published:** 2016-01-19

**Authors:** Lijuan Zou, Jerome L. Packard, Zhichao Xia, Youyi Liu, Hua Shu

**Affiliations:** ^1^School of Psychology and Education, Zaozhuang UniversityZaozhuang, China; ^2^State Key Laboratory of Cognitive Neuroscience and Learning and IDG/McGovern Institute for Brain Research, Beijing Normal UniversityBeijing, China; ^3^Center for Collaboration and Innovation in Brain and Learning Sciences, Beijing Normal UniversityBeijing, China; ^4^Cognitive Science Group, Beckman Institute, University of IllinoisUrbana, IL, USA

**Keywords:** morphological processing, Chinese, spoken words, the L-IFG, brain network

## Abstract

Morphological decomposition is an important part of complex word processing. In Chinese, this requires a comprehensive consideration of phonological, orthographic and morphemic information. The left inferior frontal gyrus (L-IFG) has been implicated in this process in alphabetic languages. However, it is unclear whether the neural mechanisms underlying morphological processing in alphabetic languages would be the same in Chinese, a logographic language. To investigate the neural basis of morphological processing in Chinese compound words, an fMRI experiment was conducted using an explicit auditory morphological judgment task. Results showed the L-IFG to be a core area in Chinese morphological processing, consistent with research in alphabetic languages. Additionally, a broad network consisting of the L-MTG, the bilateral STG and the L-FG that taps phonological, orthographic, and semantic information was found to be involved. These results provide evidence that the L-IFG plays an important role in morphological processing even in languages that are typologically different.

## Introduction

Morphology concerns the internal structure of words as reflected by systematic correlations of form (orthography, phonology) and meaning (semantics). Whether morphology has an independent representation in the mental lexicon is an ongoing debate in natural language research (Seidenberg and McClelland, 1989; Taft, 1994; Feldman et al., 1995) with several decades of psycholinguistic research showing that the human cognitive system is sensitive to the morphological structure of words (Marslen-Wilson and Tyler, 1997; Frost et al., 2000; Rastle et al., 2000, 2004; Marslen-Wilson et al., 2008). For instance, previous research has consistently shown that a word (e.g., scan) is recognized faster when it is primed by a morphologically-related inflected or derived word (e.g., scans, scanner), compared to when it is primed by a visually similar but unrelated word (e.g., scandal; Bozic et al., 2007).

The psycholinguistics of morphological processing has extended from behavioral to neuroimaging research, providing neuroanatomical evidence that morphological factors are an independent principle affecting lexical organization and processing. In English, derivationally complex words are used to investigate morphological effects because the independence of form, meaning, and morphological structure can be directly distinguished in derived words. For example, a delayed repetition priming task found that morphologically related words (e.g., scan-scanner) significantly reduced activation in the left inferior frontal gyrus (L-IFG), as compared with identity (e.g., scan-scan) and orthographic form (e.g., scan-skim) conditions. The authors proposed that the IFG is involved in the segmentation of complex words based on their surface morphological structure.

The role of the IFG in morphological processing has been confirmed in several studies across different languages (Tyler et al., 2005; Bozic et al., 2007; Bick et al., 2008, 2010; Tyler and Marslen-Wilson, 2008). Most of these studies on the neural mechanisms of morphological processing have been conducted in alphabetic languages. Bick et al. (2010), using priming to examine morphological processing in Hebrew, found activation in the left middle and inferior frontal gyri to be significantly reduced when primes were morphologically related to the targets. In another study, the same group explored neural correlates with an explicit morphological judgment task and found specific involvement of the left middle frontal gyrus and inferior parietal gyrus, with increased activation in the morphological conditions relative to semantic relatedness, rhyming, and orthographic similarity. Similarly, stronger activation in the IFG was found for inflected words compared with morphologically simple words during lexical decision (Lehtonen et al., 2006). However, since these studies were conducted using alphabetic languages and focused on processing with explicit morphological marking, it is unclear whether those findings can be generalized to a logographic language like Chinese that has a non-alphabetic orthography and only infrequently marks morphological structure explicitly.

Chinese has relatively limited inflection and derivation (in contrast to morphology-rich languages like Finnish). There are some Chinese morphemes that arguably serve similar purposes—for example, the morphemes “
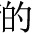
” “
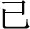
” can be added to verbs and nouns to create adjectives (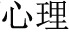-psychology becomes 
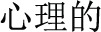
-psychological) or past participles (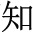-know becomes 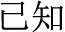-known). However, the present study emphasizes compound words composed of two separate morphemes, not including bound morphemes. More than 85% of Chinese words are complex words consisting of morphemes that contain little explicit functional marking. Morphemes correspond to a specific character in most Chinese compound words. No space exists between characters, so morpheme boundaries are sharply defined, and so there is generally no question in speaking or reading where one morpheme ends and another one begins (unlike in, e.g., English, where it is difficult to tell whether the plural morpheme in the word *places* is considered –s or –es). The fact that Chinese explicitly marks morphological structure infrequently does not mean that Chinese has no morphological structure or that morphology does not play an important role in the Chinese mental lexicon. In fact, morphological structure may be even more important in reading Chinese than in other languages, for several reasons. First, Chinese graphemes represent morphemes, and so unlike in alphabetic orthographies where graphemes generally represent phonemes, in Chinese the morpheme serves as the primary unit of interface between the written and spoken language. Second, research on child reading acquisition has shown that morphological awareness is more important than phonological awareness in learning to read Chinese, more so than in learning to read alphabetic orthographies (McBride-Chang et al., 2003; Wu et al., 2009). Third, the majority of morphemes in Chinese words are in fact ambiguous when they are presented orally—that is, most morphemes are represented by a syllable that refers to more than one morpheme. As an example, the syllable “yi4” corresponds with several different characters representing different morphemes (e.g., 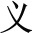 significance, 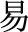 easy, 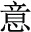 meaning). As another example, the character 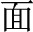 pronounced “mian4” is ambiguous, because it is a single character with an invariant pronunciation that can stand for several different morphemes in Chinese, including “flour,” “noodle,” “face,” and “surface.” Phonology, orthography, and meaning are also important in reading alphabetic languages, but in alphabetic languages morphological complexity usually is formally marked in phonology and orthography (e.g., the case of English *-ed, -s, -ing*). Also, alphabetic languages generally have greater morphological complexity, with greater numbers of inflectional and derivational affixes. These differences between Chinese and alphabetic languages in the phonology, orthography and semantics of morphological processing naturally raise the question of whether the neural mechanisms that have been found to underlie morphological processing in alphabetic languages also underlie morphological processing in Chinese.

Since there is very little explicit marking—orthographic, phonological, morphological or otherwise—indicating precisely which morpheme a syllable (e.g., /mian4/) represents, the identity of individual morphemes in Chinese may be difficult to distinguish. The way a native speaker knows which of the several possible morphemic meanings the syllable /mian4/ is referring to depends upon its occurrence within the context of the compound word. When a compound word is presented orally, multiple candidate morphemic representations may be activated during the appearance of the first syllable. As the acoustic signal unfolds and the second syllable is perceived, contextual information is provided that allows the correct morpheme to be identified (Packard, 1999). For this reason, spoken words provide a reliable way of investigating morphological effects in Chinese. For example, after the syllables /mian4-bao1/ (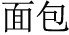, meaning “bread”) and /mian4-kong3/ (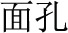, meaning “countenance”) are presented, listeners know the syllable /mian4/ means “flour” and “face” in /mian4-bao1/ and /mian4kong3/, respectively. This example shows that the meaning of a Chinese morpheme is critically dependent upon the meaning of the whole word in which it occurs, and that reading Chinese requires a comprehensive consideration of phonological (P), orthographic (O), and morphemic (M) information.

The present study aimed to investigate the neural basis of morphological processing—especially the role of IFG—in Chinese, by manipulating the first syllables of word pairs and asking native speakers to judge the same/different morphemic status of those initial syllables. Because there is a lack of comparable studies examining this phenomenon, the initial step was to design a task which can robustly activate morphological processing. There are several existing paradigms to do so, including priming as mentioned by the review of previous literature. However, effects may be subtle, and detection might require other unknown specific settings of the parameters. On the other hand, Liu et al. (2013) adopted an explicit task to determine the effect of morphemic meaning on whole word semantic access. The current study uses the same task but focuses on morphemic analysis directly. Three critical morphological conditions were created. In the P+O+M+ condition the word pairs, e.g., /gao1-wen1/ (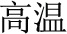, meaning high temperature) and /gao1-kong1/ (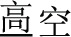, meaning high sky) share the same initial syllable /gao1/ (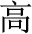, meaning “high”) with the same pronunciation (P+), the same orthography (O+), and the same morpheme (M+). In this condition, because the word pairs are semantically unrelated at the whole word level but share morphemic meaning at the individual morpheme level, there is a potential conflict in making a morphological judgment that involves both individual and whole-word semantics. In the P+O+M- condition, the initial syllable, e.g., /mian4/ of the two words in the word pair /mian4-bao1/ (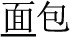, meaning bread) and /mian4-kong3/ (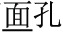, meaning face) is pronounced the same (P+) and shares the same orthography (O+), but does not represent the same morpheme (M-) in the two words (it represents “flour” in 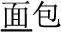 and “face” in 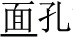). In the third condition (P+O-M-), the initial syllable, e.g., /deng1/ in the word pair /deng1guang1/ (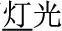, meaning “illumination”) and /deng1shan1/ (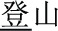, meaning “mountain climbing”) means “light” in /deng1guang1/ but “climb” in /deng1shan1/, and so the two initial syllables have the same sound (P+) but are written differently (O−) and represent two different morphemes (M−). The P+O-M- condition is expected to be the easiest, because participants can rely on the orthographic cue (i.e., the fact that they are different characters) in making the morphological judgment.

We assumed that Chinese native speaker participants would experience increased morphological processing and therefore greater neurological activation in brain areas that involve morphological processing when the morphological processing task is more difficult. Our research hypotheses specifically involving morphological processing were that additional morphological analysis would be required for the three morphological conditions relative to the Identical condition (P+O+M+ vs. Identity; P+O+M- vs. Identity; P+O-M- vs. Identity), and that therefore any brain regions that are more highly activated under the morphological conditions compared to the Identical condition would be considered to be involved in morphological processing. Using the Identical condition as a baseline does not imply an absence of morphological processing in this condition. Since morphemic processing is an automatized component in spoken word recognition, we hypothesized that it exists in all our experimental conditions. It was assumed that “yes” responses over Identical trials would be easier along with significantly less activation, while the analytical demands would be higher in other conditions. The order of morphological processing demand from high to low is expected to be P+O+M+, P+O+M-, P+O-M-, and Identity. We expected to find that when asked to judge sameness between initial morphemes in semantically unrelated spoken complex word pairs, participants would find evaluating pairs in the P+O+M+ condition to be the most difficult, resulting in the greatest amount of neural activation in the IFG. The P+O+M- condition is expected to be easier, with less IFG activation, because the characters are the same but the morphemes are different. Finally, the P+O-M- condition should involve the smallest degree of morphological processing and therefore the least amount of IFG activation compared to baseline, because both the characters and morphemes are different, and so participants might be able to rely upon both visual-orthographic and morphemic information, thereby facilitating morphological processing.

## Methods

### Participants

Seventeen college students (mean age: 21.24 years old, *SD* = 1.75, 10 females) were recruited. All participants were right-handed native speakers of Chinese with normal hearing and no reports of neurological disorders. Informed consent was obtained from all participants before the experiment, with the protocol approved by the local ethics committee.

### Stimuli

According to the P, O, and M relations between the first syllables in pairs of words, three conditions were implemented: P+O+M+ [e.g., /gao1-wen1/(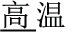 meaning high-temperature) and /gao1-kong1/ (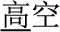, meaning high-sky)], P+O+M- [e.g., /mian4-bao1/(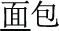, meaning bread) and /mian4-kong3(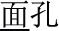, meaning face)], and P+O-M- [/deng1guang1/ (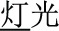, meaning “illumination”) and /deng1shan1/ (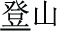, meaning “mountain climbing”)]. One hundred twenty word pairs were selected, divided into the three morphological conditions described above (P+O+M+, P+O+M-, P+O-M-). A fourth, “Identical” condition was also included as a high-level baseline. For example, the word /hai3dai4/ (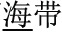, meaning “segment of sea”), served as both the original and comparison word in the Identical condition. All pairs of words in the P+O+M+, P+O+M-, and P+O-M- conditions were semantically unrelated: the semantic rating scores on a seven- point Likert scale (1 = “not related at all,” and 7 = “closely related”) were 1.84 (*SD* = 0.38) for P+O+M+, 1.18 (*SD* = 0.22) for P+O+M-, and 1.24 (*SD* = 0.21) for P+O-M-. The mean duration of the first syllable in each condition was 716 ms (*SD* = 51.5) for P+O+M+, 709 ms (*SD* = 42.8) for P+O+M-, 704 ms (*SD* = 38.6) for P+O-M-, and 715 ms (*SD* = 39.6) for Identity. The mean duration of targets in each condition was 717 ms (*SD* = 50.9) for P+O+M+, 704 ms (*SD* = 40.1) for P+O+M-, 705 ms (*SD* = 52.6) for P+O-M-, and 715 ms (*SD* = 39.6) for Identity. There was no significant difference in stimulus duration across conditions for either first (*p* = 0.51) or second words (*p* = 0.46). In addition, thirty pairs of pure tones used as a same/different baseline had values of 400 and 600 Hz for the two different tones. All speech stimuli were recorded by a female, native Mandarin speaker with a standard Beijing accent in a soundproof room on a digital audio recorder (Yamaha MG124C) using a CME MG-900 microphone at a sampling rate of 48 kHz with 16-bit resolution.

Word pairs in each of the four conditions were matched as follows: the frequency of the first syllable in first and second words [*F*_(3, 120)_ = 0.406, *p* = 0.749], the phonological family size of the first syllable in first and second words [*F*_(3, 120)_ = 0.763, *p* = 0.517], the whole word frequency of the first word [*F*_(3, 120)_ = 0.316, *p* = 0.813], the whole-word frequency of the second word [*F*_(3, 120)_ = 0.125, *p* = 0.945], the first character frequency of the first word [*F*_(3, 120)_ = 1.611, *p* = 0.191], the first character frequency of the second word [*F*_(3, 120)_ = 1.33, *p* = 0.268], the first character strokes of the first word [*F*_(3, 120)_ = 0.927, *p* = 0.430], the first character strokes of the second word [*F*_(3, 120)_ = 0.364, *p* = 0.779], the second character strokes of the first word [*F*_(3, 120)_ = 0.370, *p* = 0.775], and the second character strokes of the second word [*F*_(3, 120)_ = 0.959, *p* = 0.415]. See Supplementary Table 1 for details. All the stimuli are presented in Supplementary Table 2.

### Procedure

The auditory stimuli were presented via earphones compatible with fMRI. Before beginning the experiment, we modulated the sound volume to be clear and comfortable while the fMRI machine was operating. During the experiment, participants could adjust the volume according to their preference. We used SereneSound for fMRI from Resonance Technology Inc. (http://www.mrivideo.com/audio-stimulation.php) to deliver the experimental stimuli. The headset can reduce 30 dB gradient noise. Additionally, we used 3M™ uncorded foam earplugs. Since the earplug could further reduce the MRI environmental noise while the experimental sound volume could be adjusted by the tester, we were able to get a good SNR (signal/noise ratio), under which all the participants reported that they could hear the experimental stimuli clearly. Participants were required to judge whether the first syllable of two auditory complex words represented the same morpheme or not. The correct responses for participants were “yes” for P+O+M+ and Identical pairs, “no” for P+O+M- and P+O-M- pairs. The fMRI data for the four conditions including both “yes” and “no” responses were directly compared. It was notable that “no” trials elicited the same morphological manipulation as the “yes” trials, though “no” responses included a more complicated decision than “yes” responses. To address this issue, we included reaction time as a covariate in the statistical model and tested whether the main effect still existed. For the pure tone condition, participants were asked to judge whether two pure tones presented in succession were the same or different. The pure tone pairs and word pairs were presented randomly. Subjects responded with the right index finger for “yes” and with the right middle finger for “no.” Participants were asked to focus on the fixation cross when it appeared in the center of the screen during the intertrial interval with no response required. The four conditions (P+O+M+, P+O+M-, P+O-M-, and Identical) were presented in pseudorandom order, with a fixation cross (+) presented during a variable intertrial interval to enable fMRI jittering in an event-related design.

The experiment was divided into three runs, each containing 50 trials (40 pairs of words, 10 in each of the four morphological conditions and 10 pairs of pure tones), with each run lasting about 7 min and 40 s. Each trial consisted of presentation of the first auditory word/pure tone for 800 ms along with a blank screen, then presentation of a 200-ms blank screen, followed by the second 800-ms auditory word/pure tone also along with a blank screen, followed by a 3200-ms blank screen during which participants gave their response. Twenty practice trials were conducted before participants entered the scanner and were not included in the data analysis.

Behavioral performance was assessed during the fMRI scan, with participants viewing the fixation cross on the screen via a tilted mirror. The variable intertrial interval range (ranging from 0 to 8 s) and pseudorandom ordering was implemented using the optseq2 program (http://surfer.nmr.mgh.harvard.edu/optseq/) to achieve optimal experimental efficiency (Dale, 1999).

### Imaging parameters

Scans were acquired with a 3T Siemens Trio Scanner. An echo planar imaging (EPI) sequence was used for functional imaging with the following parameters: *TR* = 2500 ms, *TE* = 30 ms, flip angle = 90°, imaging matrix = 64 × 64, FOV = 200 mm, voxel size = 3.125 × 3.125 × 3, and 42 slices, 3 mm each with 0.485 mm gap between slices. Slices were arranged obliquely to cover most of the brain. The parameters for anatomical images were as follows: MPRAGE (Magnetization Prepared Rapid Gradient Echo) sequence, *TR* = 2530 ms, *TE* = 3.45 ms, flip angle = 7°, FOV = 256 mm, matrix = 256 × 256, slice thickness = 1 mm, voxel size = 1.0 × 1.0 × 1.3 mm, number of slices = 176.

### Data analysis

SPM5 (www.fil.ion.ucl.ac.uk/spm/) was used for image preprocessing and subsequent statistical analysis. All functional images were spatially realigned and co-registered with their corresponding anatomical images. The resulting images were then normalized to the Montreal Neurological Institute (MNI) space with transformation parameters obtained from the segmentation of the anatomical images, and smoothed with an isotropic 6-mm full-width at half-maximum (FWHM) Gaussian kernel. The resulting images were then resampled at a spatial resolution of 3 × 3 × 3 mm^3^. The time series for each voxel was high-pass filtered with a 1/128 Hz cut-off to remove low-frequency noise and signal drift. The preprocessed images were analyzed using the general linear model for each participant at the voxel-based level across the entire brain in order to calculate the effects of the experimental conditions, with a reference boxcar function of stimuli, which was convolved with a canonical hemodynamic response function. The second level (group) analysis was then performed to assess the mean brain activation for all participants using random-effects analysis. One-sample *t*-tests were conducted to determine the task effect relative to the pure tone baseline. Conjunction analysis was further used to reveal the common neural basis for the morphological conditions (P+O+M+, P+O+M-, P+O-M-).

Next, the beta value of Regions of Interest (ROI) corresponding to the morphological conditions were defined by the conjunction analysis results extracted in the P+O+M+, P+O+M-, P+O-M-, and Identical conditions. The ROIs were created in two steps. Firstly, the activation cluster was extracted during the conjunction analysis. Secondly, these clusters were intersected with the standard anatomical structure template from AAL (Tzourio-Mazoyer et al., 2002). The beta-values for all voxels in the ROI were extracted and averaged, in order to calculate the differences among the four morphological conditions. An AlphaSim corrected *p* threshold of 0.05 (combination of voxel level at 0.005 uncorrected and a cluster threshold of 729 voxels determined by a Monte Carlo simulation) was chosen for all the conditions relative to baseline.

In order to confirm the reliability of the results from conjunction analysis, one-way ANOVA within-subject in SPM was conducted using P+O+M+, P+O+M-, P+O-M-, and Identical as the four levels. The main effect results of ANOVA were used to confirm specific morphological regions that were discovered in the prior conjunction analysis. We created a 6-mm-radius sphere centered at the local peak voxels of the main effect to extract brain beta value in the P+O+M+, P+O+M-, P+O-M-, and Identical conditions. FWE (Family Wise Error) correction (*p* < 0.05) was chosen for the ANOVA analysis.

## Results

### Behavioral results

**Figure 1:**
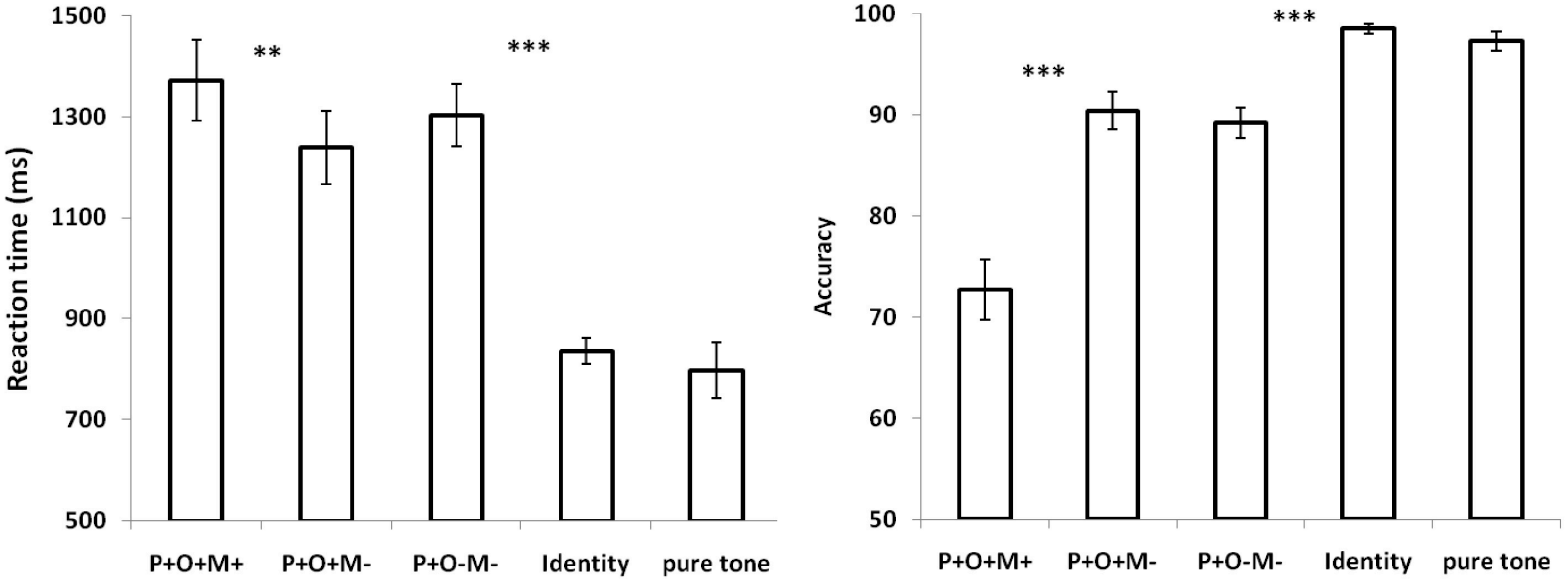
**Reaction time (RT) and accuracy during morphological processing**. Both RT and accuracy are significantly influenced by morphological conditions. Error bar = S.E.M. ^**^p < 0.01; ^***^p < 0.0005.

Response times and accuracy scores were entered into repeated-measures ANOVAs with the four morphological conditions as independent variables. In the analysis, we first discarded the RTs that were greater than three *SD*s beyond the global mean (0.98%).Then, only RTs with correct responses (91%) were included in the subsequent analysis. For RTs, the ANOVA showed a main effect of Condition (*F*_(4, 160)_ = 68.79, *p* < 0.005). Paired *t* tests revealed that the RT for the P+O+M+ was significantly longer than the P+O+M- (*t*_16_ = 2.99, *p* = 0.009), the Identical (*t*_16_ = 12.08, *p* < 0.0005) and the pure tone baselines (*t*_16_ = 14.91, *p* < 0.0005), with no difference found between the P+O+M+ and P+O-M- conditions (*p* > 0.05). This result indicates that participants took longer to judge the initial morphemes in the P+O+M+ condition. The accuracy analysis also showed a significant main effect of Condition (*F*_(4, 160)_ = 40.02, *p* < 0.005), with Paired *t* tests revealing that the accuracy for P+O+M+ was significantly lower than the P+O+M- (*t*_16_ = −5.24, *p* < 0.0005), the P+O-M-(*t*_16_ = −4.78, *p* < 0.0005), the Identical (*t*_16_ = −8.93, *p* < 0.0005) and the pure tone baseline conditions (*t*_16_ = −5.8, *p* < 0.0005). There was no difference between P+O+M- and P+O-M- (*p* > 0.05) (Figure 1).

### Imaging results

#### Overall brain activation

**Figure 2:**
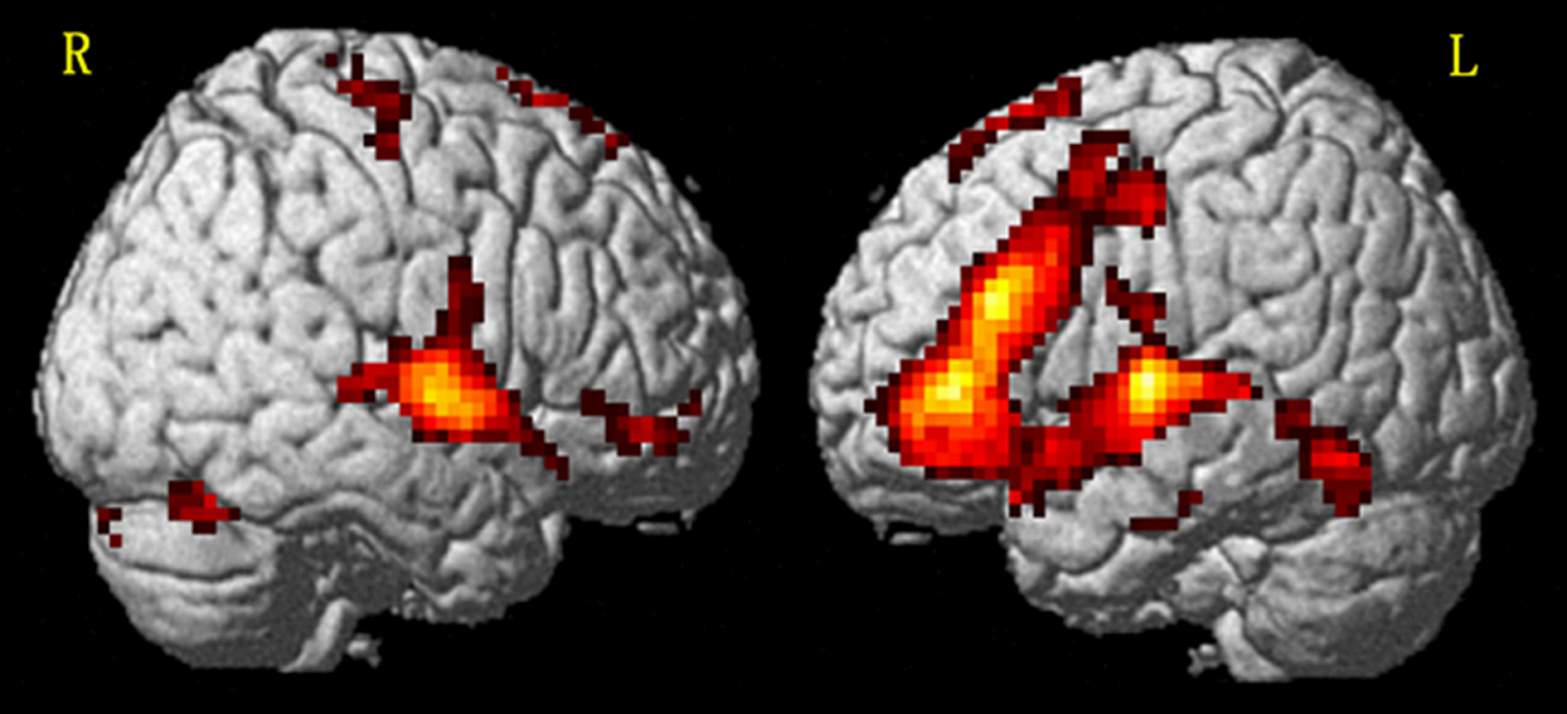
**Overall activation for speech processing relative to pure tone using random effect GLM analysis (*p* < 0.05, cluster size corrected)**. L, left hemisphere; R, right hemisphere.

We were interested in the overall brain activation that occurred while participants analyzed the Chinese complex words, and so the P+O+M+, P+O+M-, P+O-M-, and Identical conditions were combined into a general auditory word condition to be contrasted with the pure tone condition. These results showed that Chinese spoken word processing is associated with broad brain networks covering the frontal-temporal-occipital regions (Figure 2), consistent with previous findings for spoken word processing (Hickok and Poeppel, 2007; Hagoort, 2008; Moore et al., 2008; Obleser et al., 2008; Tyler and Marslen-Wilson, 2008; Price, 2010).

#### Neural correlates of morphological processing

**Figure 3:**
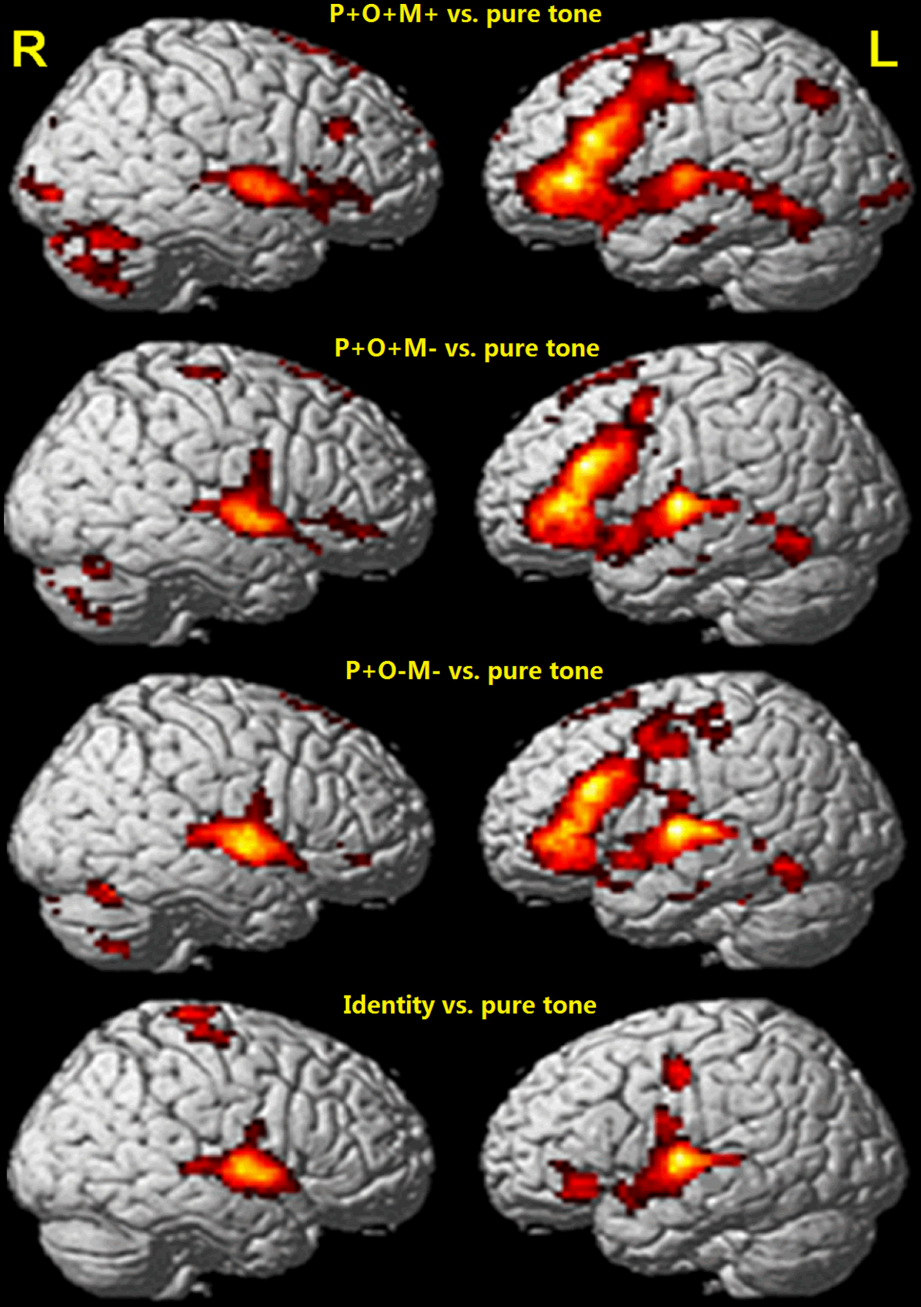
**Whole brain analysis for each condition relative to baseline (*p* < 0.05, cluster size corrected)**. L, left hemisphere; R, right hemisphere.

Relative to the pure tone baseline, each morphological condition showed a similar brain activation pattern that included bilateral frontal-temporal-parietal ROIs (Figure 3, Table 1), specifically: the L-IFG, the bilateral superior temporal gyri, the left middle temporal gyrus, the bilateral supplementary motor areas and the cerebellum (Table 1). In addition, the activation patterns also reflected the difference among morphological conditions. Overall, the distribution and intensity of BOLD signals did not significantly differ over the three morphological conditions relative to the pure tone baseline (Figure 3; *p* < 0.05, cluster size corrected). Most notably, the L-IFG specifically was involved in the three morphological conditions when compared with the Identical condition (*p* < 0.05, cluster size corrected). In order to examine whether the L-IFG was robustly activated for Chinese morphological processing, conjunction and ROI methods were used in the subsequent analysis.

**Table 1:** **Brain activation for the morphological conditions relative to the pure tone**.

**Brain area**	**BA**	**x**	**y**	**z**	***T***
**P**+**O**+**M**+ **vs. PURE TONE**					
***L-Frontal-Inf-Tri***	***45***	*−**45***	***36***	***3***	***13.85***
R-Insula	47	33	27	6	10.93
L-Supp-Motor-Area	32	−3	18	48	9.88
R-Cerebelum-Crus1		12	−75	−30	9.55
L-Frontal-Mid-Orb	45	−45	48	0	8.91
R-Temporal-Sup	48	60	−3	−3	8.22
L-Temporal-Sup	48	−60	−9	3	8.03
R-Calcarine	17	12	−66	9	7.68
L-Calcarine	17	−6	−63	9	7.18
L-Cerebelum-Crus2		−5	−81	−24	6.29
R-Occipital-Inf	18	30	−90	−3	6.22
L-Parietal-Inf	7	−30	−66	42	5.90
L-Fusiform	37	−36	−32	−19	5.72
Vermis-4-5		0	−51	−15	4.90
R-Frontal-Inf-Orb	47	33	39	−9	4.65
L-Temporal-Mid	21	−66	−30	3	4.44
L-Frontal-Sup-Medial	10	−6	66	27	4.27
L-Rectus	11	0	39	−15	4.26
L-Occipital-Inf	19	−33	−87	−9	4.15
R-Frontal-Inf	48	45	27	30	3.58
**P**+**O**+**M- vs. PURE TONE**					
***L-Frontal-Inf-Tri***	***45***	*−**45***	***36***	***3***	***9.95***
L-Temporal-Sup	22	−60	−12	6	9.52
R-Temporal-Sup	48	60	−3	−3	9.31
L-Hippocampus	20	−27	−9	−18	7.34
L-Temporal-Inf	37	−45	−51	−12	7.20
R-Rolandic-Oper	48	60	0	12	6.54
L-Supp-Motor-Area	8	−6	21	48	6.02
R-Cerebelum-Crus1		9	−81	−24	5.68
R-Cerebelum-Crus2		12	−84	−36	5.57
R-Heschl	48	68	−15	9	4.86
Vermis-9		0	−48	−36	4.75
L-Temporal-Mid	21	−66	−30	3	4.49
R-Frontal-Inf-Orb	47	36	39	−6	4.49
R-Precentral	4	39	−27	69	4.22
L-Fusiform	37	−36	−32	−19	3.92
**P**+**O-M- vs. PURE TONE**					
L-Temporal-Sup	22	−60	−9	6	8.88
L-Heschl	48	−39	−24	12	5.64
L-Temporal-Inf	20	−42	−15	−27	8.54
L-Fusiform	20	−36	−30	−18	5.06
R-Temporal-Sup	48	60	−3	−3	8.43
R-Heschl	48	48	−15	12	7.41
L-Frontal-Inf-Tri	48	−45	24	24	8.43
***L-Frontal-Inf-Tri***	***45***	*−**51***	***36***	***9***	***6.99***
L-Frontal-Mid-Orb	46	−48	48	0	6.55
R-Cerebelum-6	19	21	−60	−24	7.90
L-Temporal-Inf	37	−48	−54	−12	7.04
L-Temporal-Mid	21	−51	−45	−3	3.92
L-Supp-Motor-Area	6	−3	15	57	6.65
L-Putamen		−21	−3	9	3.73
L-Thalamus		−12	−18	9	5.43
Vermis-8		3	−60	−36	5.29
R-Frontal-Inf-Orb	47	39	42	−9	3.61
L-Cerebelum-6	37	−27	−51	−30	4.71
L-Cerebelum-4/5	37	−30	−39	−24	4.41
L-Cerebelum-8		30	−63	−48	4.39
L-Temporal-Sup	22	−60	−12	6	8.51
L-Fusiform	37	−37	−31	−18	4.51
**IDENTITY vs. PURE TONE**					
L-Temporal-Mid	22	−63	−9	0	11.33
R-Temporal-Sup	48	60	0	0	9.68
L-Temporal-Sup	48	−51	−15	3	8.97
R-Heschl	48	45	−18	6	5.58
R-Postcentral	4	18	−30	78	4.46
L-Frontal-Inf-Orb	47	−39	30	−12	4.43
L-Postcentral	43	−60	−5	21	4.37
R-Precentral	6	24	−24	75	4.35
R-Temporal-Mid	21	69	−27	0	3.94

*L, left hemisphere; R, right hemisphere; BA, Brodmann area; x, y, z = MNI Coordinate; T, T-value; voxel level threshold is 0.005 and corrected to 0.05 at cluter level. Bold characters mean contrasts between experimental conditions. Italic characters mean the common and key brain area found in each contrast*.

**Figure 4:**
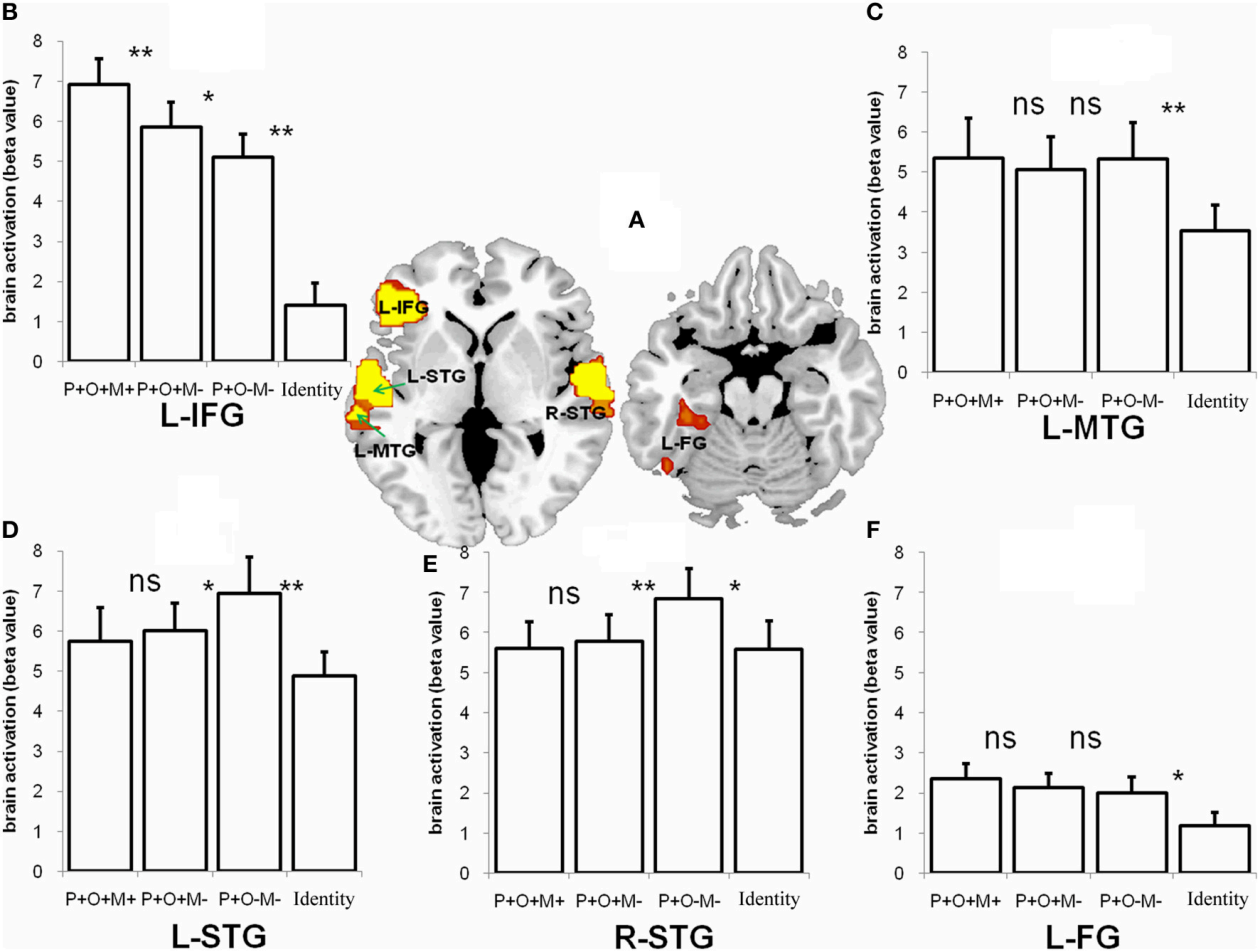
**The conjunction activation of the P+O+M+, the P+O+M-, and the P+O-M- during morphological processing and the retest of these ROIs in the Identical condition**. **(A)** The five regions including the L-IFG, the L-MTG, the L-STG, the R-STG, and the L-FG were significantly activated in the conjunction analysis (*p* < 0.05, cluster size corrected) during morphological processing **(B–F)**. The retests of these conjunctional regions in the four conditions (the P+O+M+, the P+O+M-, the P+O-M-, and the Identical condition). ns, non significant; ^**^p < 0.01; ^*^p < 0.05.

The conjunction analysis was conducted for the P+O+M+, P+O+M-, and P+O-M- conditions. A hypothesis was that comparable brain regions should be involved in processing the morphological conditions represented by the P+O+M+, P+O+M-, and P+O-M-, with P+O+M+ expected to show more activation than P+O+M- and P+O-M-. Our analysis revealed that multiple, overlapping brain regions were indeed involved in Chinese morphological processing (the threshold at voxel level is 0.005 and corrected to 0.05 at cluster level) (Figure 4A, Table 2), namely: the L-IFG, the bilateral superior temporal gyri (L-STG, R-STG), the left middle temporal gyrus (L-MTG), and the left fusiform gyrus (L-FG). These results suggest that Chinese morphological processing requires a sizable neurological network involving multiple brain regions.

**Table 2:** **Conjunction results of the P+O+M+, the P+O+M-, and the P+O-M- relative to the baseline in morphological processing**.

**Brain area**	**BA**	**Cluster size**	**x**	**y**	**z**	***T***
R_Temporal_Sup	48	275	60	−3	−3	8.22
L_Temporal_Sup	48	1462	−60	−9	3	8.03
L_Frontal-Inf_Tri	45	1462	−45	36	3	7.16
L_Temporal_Mid	21	1462	−66	−30	3	4.44
L_Fusiform	37	164	−36	−32	−19	5.72

*L, left hemisphere; R, right hemisphere; Sup, superior; Inf, inferior; Mid, middle; BA, Brodmann area; x, y, z = MNI Coordinate; T, T-value; voxel level threshold is 0.005 and corrected to 0.05 at cluter level*.

Given our interest in determining whether there are ROIs specific to morphological processing, we compared the Identical condition to the other three morphological conditions within the ROIs that showed morphological activation. The results showed that the activation pattern of the Identical condition significantly differed from the other three morphological conditions (Figure 4). Specifically, we compared morphological conditions (P+O+M+, P+O+M-, P+O-M-) with Identical condition, and comparisons across morphological conditions (P+O+M+ vs. P+O+M-, P+O+M- vs. P+O-M-) in these ROIs, as shown in Figures 4B–F. For the L-IFG, activation in the Identical condition was significantly reduced relative to the other three morphological conditions (Identical vs. P+O+M+: *t*_16_ = −13.44, *p* < 0.0005; Identical vs. P+O+M-: *t*_16_ = −9.44, *p* < 0.0005; Identical vs. P+O-M-: *t*_16_ = −2.13, *p* = 0.049), and difference between the three morphological conditions was significant (P+O+M+ vs. P+O+M-: *t*_16_ = −2.97, *p* = 0.009; P+O+M- vs. P+O-M-: *t*_16_ = 2.13, *p* = 0.049). For the L-MTG, the comparisons between the Identical and morphological conditions were significant (Identical vs. P+O+M+: *t*_16_ = −3.06, *p* = 0.007; Identical vs. P+O+M-: *t*_16_ = −3.85, *p* = 0.001; Identical vs. P+O-M-: *t*_16_ = −3.05, *p* = 0.008), with no significant difference across morphological conditions (P+O+M+ vs. P+O+M-: non-significant; P+O+M- vs. P+O-M-: non-significant). For R-STG, only comparisons between Identical and P+O-M- (Identical vs. P+O-M-: *t*_16_ = −2.48, *p* = 0.025), and between P+O+M- and P+O-M- were significant (P+O+M- vs. P+O-M-: *t*_16_ = −3.33, *p* = 0.004), the other comparisons did not reach significance (Identical vs. P+O+M+: non-significant; Identical vs. P+O+M-: non-significant); P+O+M+ vs. P+O+M-: non-significant). For L-STG, comparisons among Identical, P+O+M-, and P+O-M- reached significance (Identical vs. P+O+M-: *t*_16_ = −2.81, *p* = 0.013, Identical vs. P+O-M-: *t*_16_ = −3.83, *p* = 0.001, P+O+M- vs. P+O-M-: *t*_16_ = −2.79, *p* = 0.013). Other comparisons did not reach significance (Identical vs. P+O+M+: non-significant; P+O+M+ vs. P+O+M-: non-significant). For the FG, the comparisons between Identical and morphological conditions were significant (Identical vs. P+O+M+: *t*_16_ = −2.87, *p* = 0.011; Identical vs. P+O+M-: *t*_16_ = −2.79, *p* = 0.013; Identical vs. P+O-M-: *t*_16_ = −1.95, *p* = 0.069), with no significant difference across morphological conditions (P+O+M+ vs. P+O+M-: non-significant; P+O+M- vs. P+O-M-: non-significant).

More importantly, the three morphological conditions modulated the pattern of differences found within the L-IFG, activation from highest to lowest: P+O+M+, P+O+M-, and P+O-M- condition (Figure 4B). These findings are analogous to the behavioral results, in which the RT for the P+O+M+ condition was significantly longer than for the other two morphological conditions.

Assuming that the longer RT can be viewed as an index of general processing difficulty, in order to determine whether the activation of the L-IFG was the result of morphological processing rather than general processing difficulty, we retested the activation differences among the P+O+M+, P+O+M-, and P+O-M- conditions using RT as a covariate to partial out general processing difficulty. The result showed that the activation differences in the L-IFG were significant when RT is controlled [*F*_(2, 26)_ = 3.92, *p* < 0.04]. This result suggests that the differences in L-IFG activation may be attributed specifically to different degrees of morphological processing difficulty rather than simply general processing difficulty.

**Figure 5:**
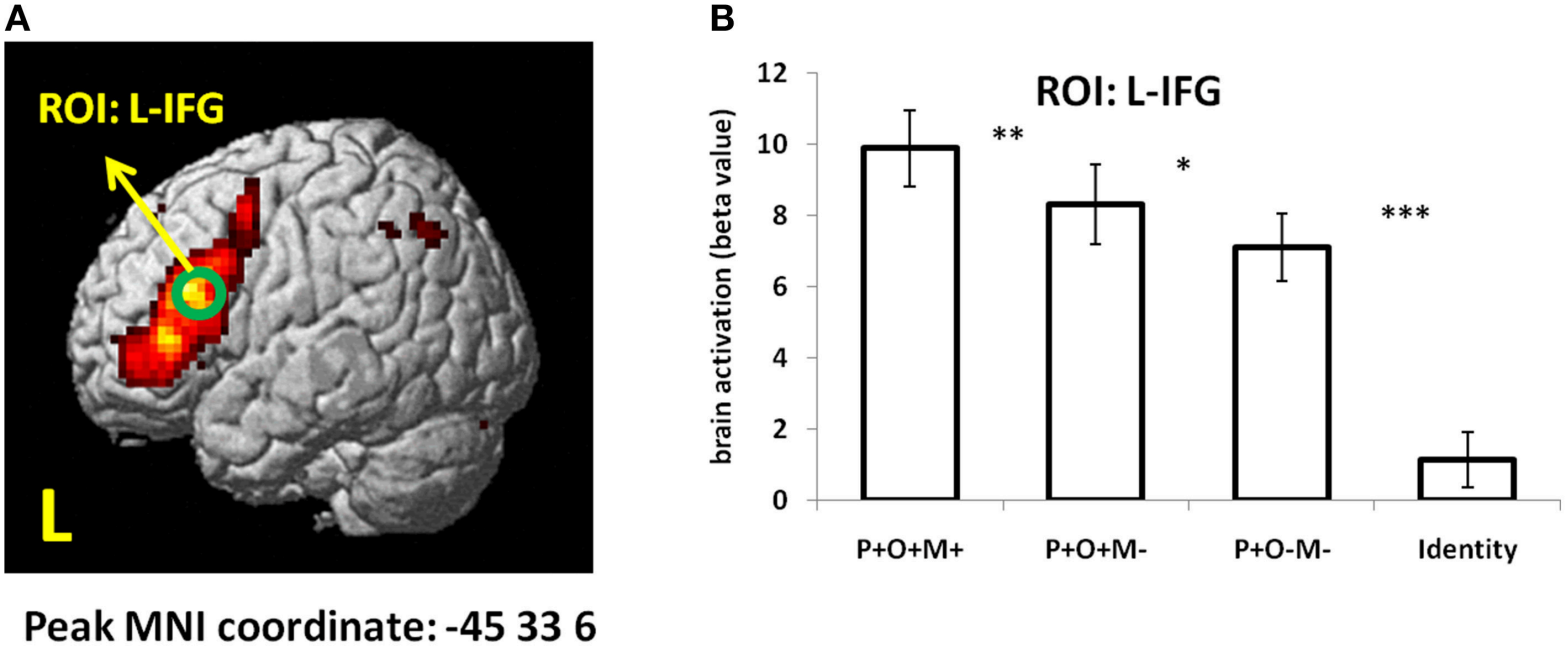
**The “One-way ANOVA—within subject” results**. In the modal, there were four levels: P+O+M+, P+O+M-, P+O-M-, and Identical. **(A)** Main effect activation map. FWE correction, *p* < 0.05. **(B)** Brain beta value in each condition. ^***^p < 0.001; ^**^p < 0.01; ^*^p < 0.05; L, left hemisphere.

To further examine whether the L-IFG found in conjunction analysis can be considered a core region in Chinese morphological processing, we checked the main effect of “One-way ANOVA—within subject” using SPM. P+O+M+, P+O+M-, P+O-M-, and Identical conditions were entered as four levels in the ANOVA analysis. Results showed that the inferior frontal gyrus (peak coordinate: –45, 33, 6, cluster size: 851 voxels) was strongly activated using a strict correction threshold (FWE corrected, *p* < 0.05) (Figure 5A). Then a 6-mm-radius sphere centering at the peak coordinate of IFG (MNI X, Y, Z: –45, 33, 6; green circle in Figure 5A) was created to extract brain beta value. Results showed that activation under the Identical condition was significantly reduced relative to the other three conditions in the L-IFG (Identical vs. P+O+M+: *t*_16_ = −12.08, *p* < 0.0005; Identical vs. P+O+M-: *t*_16_ = −8.5, *p* < 0.0005; Identical vs. P+O-M-: *t*_16_ = −7.62, *p* < 0.0005; Figure 5B). Additionally, activation in P+O+M+ was significantly larger than P+O+M- (*t*_16_ = 2.99, *p* = 0.009), and activation in P+O+M- was significantly larger than P+O-M− (*t*_16_ = 2.28, *p* = 0.037) (Figure 5B). Overall, the brain activation showed that: P+O+M+ > P+O+M- > P+O-M− > Identical (Figure 5B), which was highly consistent with the ROI results according to conjunction method (Figure 4B), indicating that the L-IFG activation found during Chinese morphological processing is a robust finding.

In addition to the L-IFG, we found that the L-MTG, the bilateral STG and the L-FG were also involved in Chinese morphological processing. However, the patterns in these regions were different from the pattern found for the L-IFG across the morphological conditions. In the P+O+M+, P+O+M-, P+O-M-, and Identical conditions, the L-MTG always showed significant activation, indicating that the mapping from phonology to semantics was rather automatic across all auditory words, although significantly less activation was found in the Identical condition than others (as seen in Figure 4C). The finding of high activation in the bilateral STG can be explained by the fact that the stimulus input was auditory. The increased activation in the P+O-M- for both the R-STG and L-STG may be attributed to lexical homophone processing (Newman, 2012). In particular, the involvement of the R-STG suggests a more bilateral processing of non-morphological phonological information. For the L-FG, the activation in the morphological conditions was greater than in the Identical condition, providing further evidence that visual-orthographic processing is involved in auditory morphological processing (Perre and Ziegler, 2008; Pattamadilok et al., 2009, 2010, 2011; Peereman et al., 2009; Perre et al., 2009a,b, 2011; Dehaene et al., 2010; Desroches et al., 2010; Zou et al., 2012).

## Discussion

The aim of the present study was to investigate the neural mechanisms underlying the morphological processing of Chinese spoken words and to determine whether such mechanisms correspond to those that have been found to underlie morphological processing in alphabetic languages. Both the behavioral and imaging results showed clearly divergent response patterns for pairs that involved more demanding morphological processing than for pairs that involved less demanding morphological processing. Behavioral results indicate that participants took longer and were less accurate in judging morphological relatedness in the P+O+M+ condition. Neuroimaging data revealed that the L-IFG was preferentially engaged under the three morphological conditions, consistent with previous findings from research on alphabetic languages. In addition, gradient IFG activation patterns corresponding to different degrees of morphological processing were found in the present study. These results provide additional evidence for the independence of morphological processing in Chinese and for neural mechanisms of morphological processing that are general and specific across languages.

Interestingly, both the behavioral and fMRI results showed that P+O+M+ was the most difficult morphological condition. This finding was not in line with the prediction according to previous priming studies that P+O+M+ should be easier and less activated than the other morphological conditions (Marslen-Wilson and Tyler, 1997; Bozic et al., 2007; Gold and Rastle, 2007; Morris et al., 2007; Lavric et al., 2011; Tsang and Chen, 2013). To better understand this result, the task paradigm adopted in the current study should be taken into account. Explicit morphological judgment requires participants to determine whether the first morpheme of two orally-presented complex words represented the same morpheme or not according to the whole-word semantics. Since Chinese morphological processing requires a comprehensive association among phonology, orthography, and whole-word semantics, participants could not distinguish morpheme meaning until they accessed the whole-word semantics. In other words, morphological judgment relied a great deal on whole-word lexical access. Pressing the “yes” button in the P+O+M+ condition entails a conflict, because the whole-word semantics are actually different. So the demand of increased morphemic processing in this condition as compared with morpheme-related processing in the other conditions can be detected by comparing this condition with the others. Moreover, we argue this effect might be bigger for skilled speakers (as in the present study), since they access whole-word meaning more automatically. Therefore, P+O+M+ elicited the highest activation in the IFG of all the morphological conditions.

Another interesting issue involves the exact role of the IFG during morphological processing. Recently, researchers have investigated the distinction between morpho-orthographic and morpho-semantic processing (Diependaele et al., 2005; Rastle and Davis, 2008), with the neuroimaging literature discussing this as well. For example, Lehtonen et al. (2006) found stronger activation in the L-IFG for inflected Finnish words compared with morphologically simple words, suggesting that the L-IFG is responsible for integrating morphemic meaning. In contrast, Bozic et al. (2007) found a decrease in L-IFG activity for morphologically related words using delayed repetition, with these authors interpreting the L-IFG activation as reflecting morpho-orthographic decomposition. Similarly, Bick et al. (2008, 2010) found that both implicit and explicit morphological processing in Hebrew significantly relied on the L-IFG, suggesting that the L-IFG may be involved in early, automatic morphological processing, which takes place during reading regardless of task. Some researchers also found involvement of the L-IFG in morphosyntactic processing, finding that the L-IFG was activated when morphology was used to retrieve gender information (Miceli et al., 2002; Hernandez et al., 2004; Heim et al., 2005; Longoni et al., 2005).

The results of the present study further contribute to the findings just discussed, in finding that morphological processing in Chinese also significantly involves the L-IFG. A difference in this work is that L-IFG activation was additionally modulated by the tripartite relationship among morphology, orthography and whole-word semantics. Specifically, the activation of the L-IFG was enhanced by words that are semantically disparate but still share a morpheme. For example, in the P+O+M+ condition exemplified by the /gao1wen1-gao1kong1/ (
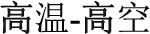
) compound word pair, the first syllable /gao1/ has the same meaning (meaning “high”), representing the same morpheme, but the meanings of the complete two words are not related (high-temperature vs. high-sky). The conflict between whole-word semantic relatedness and morpheme meaning renders it more difficult for participants to judge morphemic meaning in this condition than in the other two conditions. In contrast, there was no conflict between individual morpheme and whole-word meaning in the P+O+M- and the P+O-M- conditions, with the activation in L-IFG being significantly less for the P+O+M- and P+O-M- conditions than for the P+O+M+ condition. Furthermore, since participants could not rely on the orthographic cue in the P+O+M- condition, the activation of the L-IFG was greater in the P+O+M- relative to the P+O-M- condition, where participants were able to judge by simply relying on orthographic form. After controlling for general processing difficulty by partialing out RT, the gradient pattern for three morphological conditions in the L-IFG was still observed, strongly suggesting that the L-IFG is a key brain area serving Chinese morphological processing. Our results from skilled readers were relatively consistent with previous Chinese morphological neuroimaging results found for children. Liu et al. (Liu et al., 2013) reported that Chinese children with reading disability showed reduced activation in left dorsal posterior and ventral anterior inferior frontal gyrus compared to the typically developing children, when they made semantic relatedness judgments to incongruent word pairs that were either semantically related but did not share a morpheme or semantically unrelated but did share a morpheme. Considering the features of Chinese morphology and our specific paradigm, we speculate that the L-IFG subserves the cognitive integration of whole-word semantics and initial morpheme meaning in Chinese.

Comparably, a recent study examined the neurological basis of morphological awareness in English-speaking children with auditory input, while attempting to avoid confounding reading proficiency with children's underlying morphological ability (Arredondo et al., 2015). To achieve this goal, a morphological awareness condition, a control word-matching condition and a rest-period baseline condition were included. Results showed that during the morphological condition, children showed greater activation in the left IFG including a ventral aspect of the IFG (BA47), IFG (BA45), MFG (BA46/9), and anterior STG regions, which were associated with processing word meaning and word structure. These findings indicate that morphology tasks may engage cognitive processes important for both lexico-semantic and syntax processes, and that those regions integrate various levels of linguistic analysis, localized in and around the L-IFG (Arredondo et al., 2015). The present study is the first report that explores the structural sensitivity to morphological manipulations rather than just focusing on lexical similarity. The logic of task design in the present study was similar to Arredondo et al. (2015), while further taking advantage of the specificity of Chinese morphological processing. In sum, the L-IFG was found to be active during morphological processing in Chinese and alphabetic languages, suggesting that the functioning of this region might support key linguistic abilities necessary for learning to read and listen across languages.

In addition to the finding that the L-IFG appears to be specifically involved in Chinese morphological processing, activation in other areas during the morphological task were also found. The conjunction analysis revealed that the L-MTG, the bilateral STG, and the L-FG were all activated in the three morphological conditions (P+O+M, P+O+M-, P+O-M-). The L-MTG is a supramodal association area sensitive to both auditory and visual input (Devlin et al., 2003; Cardillo et al., 2004) that is involved in semantic processing (Binder et al., 2009; Poeppel et al., 2012). Devlin et al. (2004) found significantly reduced activation in L-MTG for word pairs with a high degree of semantic overlap during morphological processing by using the visual masked priming paradigm. In contrast, there are additional reports showing the morphological priming effect did not overlap with semantic priming (L-MTG) (Bozic et al., 2007; Gold and Rastle, 2007; Bick et al., 2008, 2010). Specifically, Gold and Rastle revealed that the priming effect was associated with the mere appearance of morphological structure (e.g., corner-CORN, Gold and Rastle, 2007). In the present study, the L-MTG was activated during our morphological judgment task, indicating that whole word semantics and initial morpheme meaning need to be integrated during Chinese morphological processing. The bilateral STG involvement may be seen as responding to phonological representation and processing (Hickok and Poeppel, 2000, 2004, 2007; Booth et al., 2002, 2004, 2006, 2008; Obleser et al., 2007, 2008; Hagoort, 2008; Price, 2010, 2012). The increased activation for the P+O-M- in the bilateral STG can be attributed to the processing of lexical homophones (Newman, 2012). The involvement of the R-STG in particular suggests a more bilateral processing of non-morphological phonological information in the lexicon. The L-FG has been found to be activated in spoken word rhyme and lexical judgment (Cone et al., 2008; Dehaene et al., 2010; Desroches et al., 2010), suggesting that orthographic processing may be automatically engaged in tasks where access to orthographic information is not required (Cone et al., 2008). Gold and Rastle (2007) revealed a partial overlap between morphological and orthographic priming. Similarly, our results found robust activation in the L-FG during Chinese auditory morphological judgment, providing additional evidence that orthographic information is involved in spoken word recognition and may be required during Chinese morphological processing.

## Conclusion

The results of this study demonstrate that Chinese morphological processing evokes a L-IFG response consistent with findings in alphabetic languages. In addition, a wide network including the L-MTG, the bilateral STG and the L-FG was activated during Chinese morphological processing. These results expand the knowledge of morphological processing as found by neuroanatomical studies of explicit morphological marking in alphabetic languages. Additional research is necessary to fully describe the network and its dynamics.

## Author contributions

LZ, HS, and YL designed research; LZ and ZX performed research; LZ and YL analyzed data; and LZ, HS, and JP wrote the paper.

### Conflict of interest statement

The authors declare that the research was conducted in the absence of any commercial or financial relationships that could be construed as a potential conflict of interest.

## References

[B1] Arredondo M. M. Ip K. I. Hsu L. S. J. Tardif T. Kovelman I. (2015). Brain bases of morphological processing in young children. Hum. Brain Mapp. 36, 2890–2900. 10.1002/hbm.22815 25930011 PMC5374976

[B2] Bick A. Goelman G. Frost R. (2008). Neural correlates of morphological processes in Hebrew. J. Cogn. Neurosci. 20, 406–420. 10.1162/jocn.2008.20028 18004948

[B3] Bick A. S. Frost R. Goelman G. (2010). Imaging implicit morphological processing: evidence from Hebrew. J. Cogn. Neurosci. 22, 1955–1969. 10.1162/jocn.2009.21357 19803693 PMC3694408

[B4] Binder J. R. Desai R. H. Graves W. W. Conant L. L. (2009). Where is the semantic system? A critical review and meta-analysis of 120 functional neuroimaging studies. Cereb. Cortex 19, 2767-2796. 10.1093/cercor/bhp055 19329570 PMC2774390

[B5] Booth J. R. Burman D. D. Meyer J. R. Gitelman D. R. Parrish T. B. Mesulam M. M. (2002). Modality independence of word comprehension. Hum. Brain Mapp. 16, 251–261. 10.1002/hbm.10054 12112766 PMC6871904

[B6] Booth J. R. Burman D. D. Meyer J. R. Gitelman D. R. Parrish T. B. Mesulam M. M. (2004). Development of brain mechanisms for processing orthographic and phonologic representations. J. Cogn. Neurosci. 16, 1234–1249. 10.1162/0898929041920496 15453976 PMC1382289

[B7] Booth J. R. Lu D. Burman D. D. Chou T. L. Jin Z. Peng D. L.. (2006). Specialization of phonological and semantic processing in Chinese word reading. Brain Res. 1071, 197–207. 10.1016/j.brainres.2005.11.097 16427033 PMC2626184

[B8] Booth J. R. Mehdiratta N. Burman D. D. Bitan T. (2008). Developmental increases in effective connectivity to brain regions involved in phonological processing during tasks with orthographic demands. Brain Res. 1189, 78–89. 10.1016/j.brainres.2007.10.080 18068690 PMC2692511

[B9] Bozic M. Marslen-Wilson W. D. Stamatakis E. A. Davis M. H. Tyler L. K. (2007). Differentiating morphology, form, and meaning: neural correlates of morphological complexity. J. Cogn. Neurosci. 19, 1464–1475. 10.1162/jocn.2007.19.9.1464 17714008

[B10] Cardillo E. Utman J. A. Matthews P. M. Devlin J. T. (2004). Left inferior prefrontal cortex activity reflects inhibitory rather than facilitatory priming. J. Cogn. Neurosci. 16, 1552–1561. 10.1162/0898929042568523 15601518 PMC2651466

[B11] Cone N. E. Burman D. D. Bitan T. Bolger D. J. Booth J. R. (2008). Developmental changes in brain regions involved in phonological and orthographic processing during spoken language processing. Neuroimage 41, 623–635. 10.1016/j.neuroimage.2008.02.055 18413290 PMC2443702

[B12] Dale A. M. (1999). Optimal experimental design for event-related fMRI. Hum. Brain Mapp. 8, 109–114. 10524601 10.1002/(SICI)1097-0193(1999)8:2/3<109::AID-HBM7>3.0.CO;2-W PMC6873302

[B13] Dehaene S. Pegado F. Braga L. W. Ventura P. Nunes Filho G. Jobert A.. (2010). How learning to read changes the cortical networks for vision and language. Science 330, 1359–1364. 10.1126/science.1194140 21071632

[B14] Desroches A. S. Cone N. E. Bolger D. J. Bitan T. Burman D. D. Booth J. R. (2010). Children with reading difficulties show differences in brain regions associated with orthographic processing during spoken language processing. Brain Res. 1356, 73–84. 10.1016/j.brainres.2010.07.097 20691675 PMC2942963

[B15] Devlin J. T. Jamison H. L. Matthews P. M. Gonnerman L. M. (2004). Morphology and the internal structure of words. Proc. Natl. Acad. Sci. U.S.A. 101, 14984–14988. 10.1073/pnas.0403766101 15358857 PMC522020

[B16] Devlin J. T. Matthews P. M. Rushworth M. F. (2003). Semantic processing in the left inferior prefrontal cortex: a combined functional magnetic resonance imaging and transcranial magnetic stimulation study. J. Cogn. Neurosci. 15, 71–84. 10.1162/089892903321107837 12590844

[B17] Diependaele K. Sandra D. Grainger J. (2005). Masked cross-modal morphological priming: unravelling morpho-orthographic and morpho-semantic influences in early word recognition. *Lang. Cogn*. Process. 20, 75–114. 10.1080/01690960444000197

[B18] Feldman L. B. Frost R. Pnini T. (1995). Decomposing words into their constituent morphemes: evidence from English and Hebrew. J. Exp. Psychol. Learn. Mem. Cogn. 21, 947–960. 7673870 10.1037//0278-7393.21.4.947

[B19] Frost R. Deutsch A. Gilboa O. Tannenbaum M. Marslen-Wilson W. (2000). Morphological priming: dissociation of phonological, semantic, and morphological factors. Mem. Cogn. 28, 1277–1288. 10.3758/BF03211828 11219955

[B20] Gold B. T. Rastle K. (2007). Neural correlates of morphological decomposition during visual word recognition. J. Cogn. Neurosci. 19, 1983–1993. 10.1162/jocn.2007.19.12.1983 17892394

[B21] Hagoort P. (2008). The fractionation of spoken language understanding by measuring electrical and magnetic brain signals. Philos. Trans. R. Soc. B Biol. Sci. 363, 1055–1069. 10.1098/rstb.2007.2159 PMC2606796 17890190

[B22] Heim S. Alter K. Friederici A. D. (2005). A dual-route account for access to grammatical gender: Evidence from functional MRI. Anat. Embryol. 210, 473–483. 10.1007/s00429-005-0032-6 16180020

[B23] Hernandez A. E. Kotz S. A. Hofmann J. Valentin V. V. Dapretto M. Bookheimer S. Y. (2004). The neural correlates of grammatical gender decisions in Spanish. Neuroreport 15, 863–866. 10.1097/00001756-200404090-00026 15073532

[B24] Hickok G. Poeppel D. (2000). Towards a functional neuroanatomy of speech perception. Trends Cogn. Sci. 4, 131–138. 10.1016/S1364-6613(00)01463-7 10740277

[B25] Hickok G. Poeppel D. (2004). Dorsal and ventral streams: a framework for understanding aspects of the functional anatomy of language. Cognition 92, 67–99. 10.1016/j.cognition.2003.10.011 15037127

[B26] Hickok G. Poeppel D. (2007). The cortical organization of speech processing. Nat. Rev. Neurosci. 8, 393–402. 10.1038/nrn2113 17431404

[B27] Lavric A. Rastle K. Clapp A. (2011). What do fully visible primes and brain potentials reveal about morphological decomposition? Neuropsychologia 48, 676–686. 10.1111/j.1469-8986.2010.01125.x 20883504

[B28] Lehtonen M. Vorobyev V. A. Hugdahl K. Tuokkola T. Laine M. (2006). Neural correlates of morphological decomposition in a morphologically rich language: an fMRI study. Brain Lang. 98, 182–193. 10.1016/j.bandl.2006.04.011 16725189

[B29] Liu L. Tao R. Wang W. You W. Peng D. Booth J. R. (2013). Chinese dyslexics show neural differences in morphological processing. Dev. Cogn. Neurosc. 6, 40–50. 10.1016/j.dcn.2013.06.004 23872198 PMC6987802

[B30] Longoni F. Grande M. Hendrich V. Kastrau F. Huber W. (2005). An fMRI study on conceptual, grammatical, and morpho-phonological processing. Brain Cogn. 57, 131–134. 10.1016/j.bandc.2004.08.032 15708203

[B31] Marslen-Wilson W. D. Bozic M. Randall B. (2008). Early decomposition in visual word recognition: dissociating morphology, form, and meaning. Lang. Cogn. Process. 23, 394–421. 10.1080/01690960701588004 18923643 PMC2557072

[B32] Marslen-Wilson W. D. Tyler L. K. (1997). Dissociating types of mental computation. Nature 387, 592–594. 9177345 10.1038/42456

[B33] McBride-Chang C. Shu H. Zhou A. B. Wat C. P. Wagner R. K. (2003). Morphological awareness uniquely predicts young children's Chinese character recognition. J. Edu. Psychol. 95, 743–751. 10.1037/0022-0663.95.4.743

[B34] Miceli G. Turriziani P. Caltagirone C. Capasso R. Tomaiuolo F. Caramazza A. (2002). The neural correlates of grammatical gender: an fMRI investigation. J. Cogn. Neurosci. 14, 618–628. 10.1162/08989290260045855 12126502

[B35] Moore B. C. Tyler L. K. Marslen-Wilson W. (2008). Introduction. The perception of speech: from sound to meaning. Philos. Trans. R. Soc. Lond. B Biol. Sci. 363, 917–921. 10.1098/rstb.2007.2195 17827100 PMC2042536

[B36] Morris J. Frank T. Grainger J. Holcomb P.. (2007). Semantic transparency and masked morphological priming: an ERP investigation. Psychophysiology 44, 506–521. 10.1111/j.1469-8986.2007.00538.x 17498223 PMC2750868

[B37] Newman S. D. (2012). The homophone effect during visual word recognition in children: an fMRI study. Psychol. Res. 76, 280–291. 10.1007/s00426-011-0347-2 21660483

[B38] Obleser J. Eisner F. Kotz S. A. (2008). Bilateral speech comprehension reflects differential sensitivity to spectral and temporal features. J. Neurosci. 28, 8116–8123. 10.1523/JNEUROSCI.1290-08.2008 18685036 PMC6670773

[B39] Obleser J. Zimmermann J. Van Meter J. Rauschecker J. P. (2007). Multiple stages of auditory speech perception reflected in event-related FMRI. Cereb. Cortex 17, 2251–2257. 10.1093/cercor/bhl133 17150986

[B40] Packard J. L. (1999). Lexical access in Chinese speech comprehension and production. Brain Lang. 68, 89–94. 10.1006/brln.1999.2102 10433744

[B41] Pattamadilok C. Knierim I. N. Kawabata Duncan K. J. Devlin J. T. (2010). How does learning to read affect speech perception? J. Neurosci. 30, 8435–8444. 10.1523/JNEUROSCI.5791-09.2010 20573891 PMC6634630

[B42] Pattamadilok C. Perre L. Dufau S. Ziegler J. C. (2009). On-line orthographic influences on spoken language in a semantic task. J. Cogn. Neurosci. 21, 169–179. 10.1162/jocn.2009.21014 18476763

[B43] Pattamadilok C. Perre L. Ziegler J. C. (2011). Beyond rhyme or reason: ERPs reveal task-specific activation of orthography on spoken language. Brain Lang. 116, 116–124. 10.1016/j.bandl.2010.12.002 21211833

[B44] Peereman R. Dufour S. Burt J. S. (2009). Orthographic influences in spoken word recognition: the consistency effect in semantic and gender categorization tasks. Psychon. Bull. Rev. 16, 363–368. 10.3758/PBR.16.2.363 19293108

[B45] Perre L. Bertrand D. Ziegler J. C. (2011). Literacy Affects Spoken Language in a non-linguistic task: an ERP study. Front. Psychol. 2:274. 10.3389/fpsyg.2011.00274 22025917 PMC3198050

[B46] Perre L. Midgley K. Ziegler J. C. (2009a). When beef primes reef more than leaf: orthographic information affects phonological priming in spoken word recognition. Psychophysiology 46, 739–746. 10.1111/j.1469-8986.2009.00813.x 19386047

[B47] Perre L. Pattamadilok C. Montant M. Ziegler J. C. (2009b). Orthographic effects in spoken language: on-line activation or phonological restructuring? Brain Res. 1275, 73–80. 10.1016/j.brainres.2009.04.018 19376099

[B48] Perre L. Ziegler J. C. (2008). On-line activation of orthography in spoken word recognition. Brain Res. 1188, 132–138. 10.1016/j.brainres.2007.10.084 18062940

[B49] Poeppel D. Emmorey K. Hickok G. Pylkkänen L. (2012). Towards a new neurobiology of language. J. Neurosci. 32, 14125–14131. 10.1523/JNEUROSCI.3244-12.2012 23055482 PMC3495005

[B50] Price C. J. (2010). The anatomy of language: a review of 100 fMRI studies published in 2009. Ann. N.Y. Acad. Sci. 1191, 62–88. 10.1111/j.1749-6632.2010.05444.x 20392276

[B51] Price C. J. (2012). A review and synthesis of the first 20 years of PET and fMRI studies of heard speech, spoken language and reading. Neuroimage 62, 816–847. 10.1016/j.neuroimage.2012.04.062 22584224 PMC3398395

[B52] Rastle K. Davis M. (2008). Morphological decomposition based on the analysis of orthography. *Lang. Cog*n. Process. 23, 7–8. 10.1080/01690960802069730

[B53] Rastle K. Davis M. H. New B. (2004). The broth in my brother's brothel: morpho-orthographic segmentation in visual word recognition. Psychon. Bull. Rev. 11, 1090–1098. 10.3758/BF03196742 15875981

[B54] Rastle K. Harrington J. Coltheart M. Palethorpe S. (2000). Reading aloud begins when the computation of phonology is complete. J. Exp. Psychol. Hum. Percept. Perform. 26, 1178–1191. 10.1037/0096-1523.26.3.1178 10884016

[B55] Seidenberg M. S. McClelland J. L. (1989). A distributed, developmental model of word recognition and naming. Psychol. Rev. 96, 523–568. 10.1037/0033-295X.96.4.523 2798649

[B56] Taft M. (1994). Interactive-activation as a framework for understanding morphological processing. Lang. Cogn. Process. 9, 271–294.

[B57] Tsang Y.-K. Chen H.-C. (2013). Early morphological processing is sensitive to morphemic meanings: evidence from processing ambiguous morphemes. J. Mem. Lang. 68, 223–239. 10.1016/j.jml.2012.11.003

[B58] Tyler L. K. Marslen-Wilson W. (2008). Fronto-temporal brain systems supporting spoken language comprehension. Phil. Trans. R. Soc. Lond. B Biol. Sci. 363, 1037–1054. 10.1098/rstb.2007.2158 17827104 PMC2494576

[B59] Tyler L. K. Marslen-Wilson W. D. Stamatakis E. A. (2005). Differentiating lexical form, meaning, and structure in the neural language system. Proc. Natl. Acad. Sci. U.S.A. 102, 8375–8380. 10.1073/pnas.0408213102 15923263 PMC1149400

[B60] Tzourio-Mazoyer N. Landeau B. Papathanassiou D. Crivello F. Etard O. Delcroix N.. (2002). Automated anatomical labeling of activations in SPM using a macroscopic anatomical parcellation of the MNI MRI single-subject brain. Neuroimage 15, 273–289. 10.1006/nimg.2001.0978 11771995

[B61] Wu X. Y. Anderson R. C. Li W. L. Wu X. C. Li H. Zhang J. (2009). Morphological awareness and Chinese children's literacy development: an intervention study. Sci. Stud. Read. 13, 26–52. 10.1080/10888430802631734

[B62] Zou L. Desroches A. S. Liu Y. Xia Z. Shu H. (2012). Orthographic facilitation in Chinese spoken word recognition: an ERP study. Brain Lang. 123, 164–173. 10.1016/j.bandl.2012.09.006 23098916

