# Supplementary material for: Neural Correlates of Morphological Processing: Evidence from Chinese
Source: Front Hum Neurosci. 2016 Jan 19;9:714. doi: 10.3389/fnhum.2015.00714 (PMC4717318; doi:10.3389/fnhum.2015.00714)
Supplement: Supplementary file 1 [file Table1.DOC]

**Supplementary Material**

**Table 1. Characteristics of each condition**

| TYPE | SRS | MRS | Prime  FSF | Target  FSF | Prime  FCPF | Target  FCPF | Prime  WF | Target  WF | Prime  FCF | Target  FCF | Prime  FNS | Target  FNS | Prime  SNS | Target  SNS |
| --- | --- | --- | --- | --- | --- | --- | --- | --- | --- | --- | --- | --- | --- | --- |
| P+O+M+ | 1.84  (0.38) | 5.73  (0.43) | 271.17  (319.57) | 271.17  (319.57) | 5.03  (2.63) | 5.03  (2.63) | 18.27  （54.13） | 11.88  (15.47) | 172.18  (282.92) | 172.18  (282.92) | 7.70  (3.03) | 7.70  (3.03) | 8.90  (3.58) | 7.97  (3.19) |
| P+O+M- | 1.18  (0.22) | 1.89  (0.34) | 238.76  (500.93) | 238.76  (500.93) | 5.50  (3.36) | 5.50  (3.36) | 12.30  （22.58） | 13.31  (23.20) | 84.84  (142.07) | 84.84  (142.07) | 7.57  (2.67) | 7.57  (2.67) | 8.40  (3.37) | 8.40  (3.21) |
| P+O-M- | 1.23（0.21） |  | 310.42  （391.58） | 310.42  （391.58） | 5.50  （2.11） | 5.50  （2.11） | 11.23  （11.35） | 10.84  (9.09) | 86.88  (157.88) | 157.49  (293.85) | 8.50  (1.89) | 7.57  (2.65) | 7.93  (3.81) | 7.20  (2.79) |
| Identity |  |  | 2204.79  (309.37) | 2204.79  (309.37) | 6.30  (4.56) | 6.30  (4.56) | 12.65  （14.97） | 12.65  （14.97） | 80.55  （141.13） | 80.55  （141.13） | 8.23  （2.28） | 8.23  （2.28） | 8.40  （3.43） | 8.40  （3.43） |
| F | 51.14 | 1462.19 | 0.41 | 0.41 | 0.76 | 0.76 | 0.32 | 0.13 | 1.61 | 1.33 | 0.93 | 0.93 | 0.37 | 0.96 |
| *p* | <0.005 | <0.005 | 0.75 | 0.75 | 0.52 | 0.52 | 0.81 | 0.95 | 0.19 | 0.27 | 0.43 | 0.43 | 0.76 | 0.42 |

Note: SRS: semantic related scores; MRS: morphological related scores; FSF: the frequency of the first syllabler per million; FCPF: the phonological family size of first syllable; WF: whole word frequency per million; FCF: the frequency of first character per million; FNS: the number of strokes of first character; SNS: the number of strokes of second character; PD: prime duration; TD: target duration. The number in the parenthesis is standard deviant. These data were collected from the Chinese lexical database (Yu et al., 1998).
