# Supplementary material for: Neural Correlates of Morphological Processing: Evidence from Chinese
Source: Front Hum Neurosci. 2016 Jan 19;9:714. doi: 10.3389/fnhum.2015.00714 (PMC4717318; doi:10.3389/fnhum.2015.00714)
Supplement: Supplementary file 2 [file Table2.DOC]

**Supplementary Material**

**Table 2.** Material used in fMRI experiment

| P+O+M+ | | P+O+M- | | P+O-M- | | Identity | |
| --- | --- | --- | --- | --- | --- | --- | --- |
| prime | target | prime | target | prime | target | prime | target |
| 按摩 | 按钮 | 被褥 | 被害 | 保镖 | 宝石 | 领取 | 领取 |
| 白发 | 白鸽 | 剧痛 | 剧组 | 比分 | 彼此 | 真情 | 真情 |
| 半年 | 半径 | 钟头 | 钟爱 | 比喻 | 笔记 | 战后 | 战后 |
| 伴侣 | 伴奏 | 节奏 | 节水 | 笔墨 | 比率 | 气流 | 气流 |
| 宝库 | 宝座 | 面包 | 面孔 | 变动 | 遍布 | 附属 | 附属 |
| 保温 | 保养 | 故乡 | 故障 | 兵器 | 冰雹 | 分批 | 分批 |
| 本土 | 本职 | 机场 | 机遇 | 病情 | 并列 | 度假 | 度假 |
| 边关 | 边际 | 开车 | 开除 | 部首 | 步枪 | 负债 | 负债 |
| 彩旗 | 彩虹 | 面具 | 面条 | 采购 | 彩色 | 选拔 | 选拔 |
| 大风 | 大门 | 花费 | 花灯 | 常温 | 长途 | 优质 | 优质 |
| 定律 | 定期 | 局长 | 局限 | 初次 | 出路 | 闲聊 | 闲聊 |
| 高空 | 高温 | 旅店 | 旅长 | 船舱 | 传说 | 总分 | 总分 |
| 黑板 | 黑人 | 庄园 | 庄重 | 船只 | 传奇 | 施肥 | 施肥 |
| 红茶 | 红星 | 运费 | 运气 | 担保 | 单独 | 家乡 | 家乡 |
| 话题 | 话筒 | 纵容 | 纵向 | 灯光 | 登门 | 落户 | 落户 |
| 黄豆 | 黄金 | 信箱 | 信仰 | 读书 | 毒品 | 热水 | 热水 |
| 火箭 | 火山 | 解雇 | 解说 | 房价 | 防御 | 奇观 | 奇观 |
| 积雪 | 积蓄 | 长相 | 长辈 | 封建 | 风雨 | 客流 | 客流 |
| 巨浪 | 巨人 | 号召 | 号码 | 股东 | 古老 | 交警 | 交警 |
| 聚餐 | 聚焦 | 空想 | 空军 | 故居 | 固体 | 哀痛 | 哀痛 |
| 绿洲 | 绿豆 | 值钱 | 值班 | 黄牛 | 皇后 | 优点 | 优点 |
| 内心 | 内战 | 开关 | 开水 | 借口 | 戒心 | 药店 | 药店 |
| 平房 | 平头 | 气色 | 气垫 | 录音 | 露天 | 放松 | 放松 |
| 全文 | 全军 | 草丛 | 草案 | 绿茶 | 律师 | 专断 | 专断 |
| 手语 | 手绢 | 情报 | 情操 | 明日 | 名声 | 延期 | 延期 |
| 特权 | 特性 | 照料 | 照搬 | 明天 | 名字 | 返回 | 返回 |
| 同期 | 同胞 | 草稿 | 草帽 | 牧草 | 木偶 | 稀少 | 稀少 |
| 乌鸦 | 乌云 | 服药 | 服气 | 诗人 | 师长 | 难听 | 难听 |
| 新闻 | 新年 | 机会 | 机械 | 羊肉 | 阳台 | 落日 | 落日 |
| 真相 | 真心 | 节能 | 节拍 | 养殖 | 氧气 | 险情 | 险情 |
